# The Correlation of Reverse Redistribution Pattern with Coronary Angiography: A Conundrum Revisited

**DOI:** 10.1055/s-0044-1789209

**Published:** 2024-08-20

**Authors:** Hakan Gökalp Uzun, Selim Ekinci, Kutluhan Eren Hazır, Mücalp Alpay

**Affiliations:** 1 Department of Cardiology, Tepecik Training and Research Hospital, İzmir, Türkiye; 2 Department of Nuclear Medicine, Tepecik Training and Research Hospital, İzmir, Türkiye

**Keywords:** coronary angiography, myocardial perfusion imaging, myocardial scintigraphy, reverse redistribution, technetium Tc 99m sestamibi

## Abstract

**Objective** The reverse redistribution (RR) pattern is a phenomenon whose etiology, pathophysiology, and clinical implications are not well understood. The studies examining this pattern date back to days when timely coronary interventions and anti-ischemic therapies were not widely used, so we aimed to reinvestigate any relationship between RR and coronary angiography (CA) findings in today's contemporary clinical settings.

**Methods** All patients with an RR pattern on the Tc99m-MIBI (technetium-99m sestamibi) scan between 2021 and 2023 were screened. Information on demographics, history of acute coronary syndrome, revascularization, comorbidities, and risk factors was collected. The CA findings were compared to RR regions. The physician's decision in the case of the RR pattern was grouped.

**Results** In a total of 67 patients (men 83%, aged 63.6 ± 10.5), the RR pattern was most commonly seen in the inferior-posterior wall ( *n* = 41, 31.3%), followed by the apex ( *n* = 19, 14.5%) and anterior ( *n* = 12, 9.2%). Most patients with RR pattern had normal/nonobstructive coronary angiograms (61.1%, *n* = 22); significant stenoses in 1, 2, and 3 vessels were present in 19.4% ( *n* = 7), 13.8% ( *n* = 5), and 5.5% ( *n* = 2) of patients, respectively. There was no correlation between the regions of the RR pattern and significant stenosis detected on CA ( *p* = 0.6, *p* = 0.5, *p* = 0.6, respectively, for left anterior descending artery, circumflex artery, and right coronary artery).

**Conclusion** In this study, no evidence of a relationship between RR patterns and CA findings was found. The 60% of the patients with RR pattern had normal/nonobstructive coronaries, so the decision to proceed with CA should not be made based solely on this finding.

## Introduction

Reverse redistribution (RR) pattern is first reported with thallium-201 (Tl-201) on delayed myocardial perfusion imaging (MPI) at rest. Although blood pool-myocardial redistribution after injection is an inherent property of Tl-201 and has been used in viability testing, it has also been reported, albeit to a lesser extent, with other radiopharmaceutical agents once thought to lack this property, such as technetium-99m sestamibi (Tc99m-MIBI) and technetium-99m tetrofosmin.

Generally, the term RR applies to two situations: (1) when tracer uptake during the stress phase is normal, but rest images show an apparent defect, or (2) when a defect present in stress images is exacerbated in rest images.

Although reported in different clinical situations such as systemic lupus erythematosus, amyloidosis, sarcoidosis, Chagas disease, cardiac syndrome X, vasospastic angina, posttransplantation, or merely as an artifact, most studies have examined RR soon after myocardial infarction (MI), 1 2 and during a period when timely coronary interventions and dual antiaggregant therapy were not widely used. So, the aim of this study was to reevaluate the RR pattern and its relationship with coronary angiogram findings in the contemporary clinical setting, where the use of more potent antithrombotic agents as well as disease-modifying drugs such as statins is common, and to guide cardiologists as to whether this finding is sufficient to proceed with invasive angiography.

## Methods

### Study Population and Inclusion/Exclusion Criteria

**Fig. 1:**
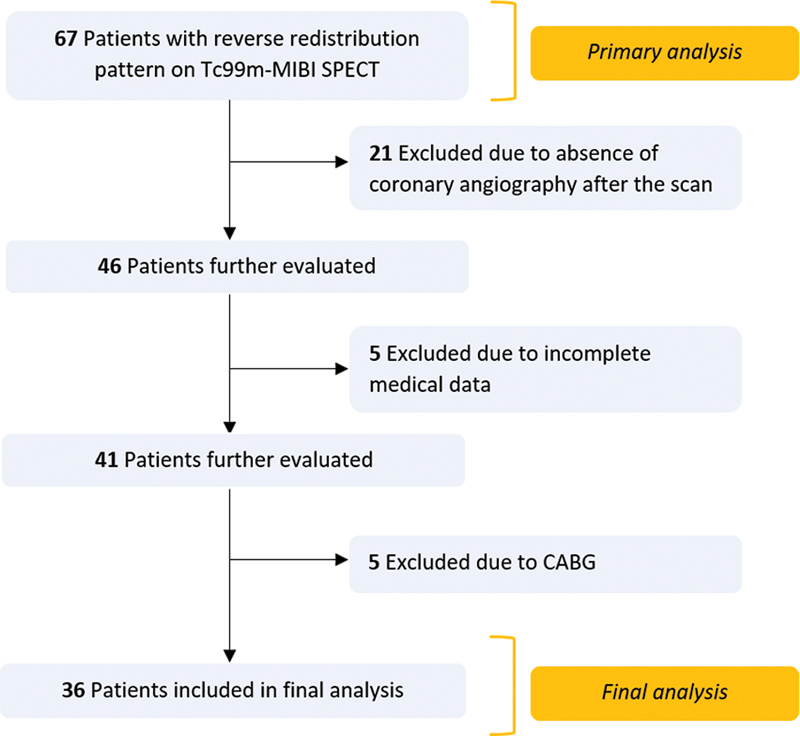
Study flowchart.

All patients with an RR pattern on Tc99m-MIBI scan done for myocardial ischemia detection between 2021 and 2023 (first 6 months) were retrospectively extracted from the hospital patient database. A total of 67 patients with RR patterns in 5,746 scans were identified (1.1%). Alongside demographic information, data about previous acute coronary syndrome (ACS; ST-segment elevation MI, non-ST elevation MI, unidentified ACS), revascularization history (type of the procedure and revascularized coronaries, if any, were also obtained), comorbidities, and risk factors (diabetes, hypertension, dyslipidemia, and smoking) were collected. The primary analysis was performed in all patients to identify population characteristics and the ventricular regions where the RR pattern was most prevalent. Then, patients with insufficient medical records ( *n* = 5), a history of coronary artery bypass grafting (CABG) ( *n* = 12), and those who did not undergo coronary angiography (CA) after the MIBI scan ( *n* = 21) were excluded from the final analysis (some patients were excluded on overlapping criteria, see study flowchart in Fig. 1 ).

The physician's decision in the case of the RR pattern was grouped as follows: medical-only treatment, invasive CA, coronary computed tomography (CT) angiography, and cardiac positron emission tomography (PET) scan.

### Image Acquisition and Analysis of Single-Photon Emission CT

MPI was performed using a single isotope, stress-only protocol on an NM830 single-photon emission CT imaging system (GE Healthcare, Chicago, Illinois, United States). Adenosine was used for pharmacologic stress in all patients. In this protocol, patients first underwent stress imaging with intravenous adenosine infusion dosed according to the patient's weight for 6 minutes. Three minutes after adenosine infusion, 8 to 10 mCi Tc99m-MIBI was administered. Intravenous aminophylline was kept ready with the patient for the possible risk of respiratory depression with adenosine. The images were processed and generated by software and interpreted by an experienced nuclear medicine specialist. If no abnormalities were seen in the stress image, the resting image was not required, and the study was considered complete. However, if the stress image was abnormal, the patient was called back on another day for the rest imaging. Stress and rest left ventricular ejection fraction (LVEF) were calculated by software when possible. RR was defined as a defect that appears or worsens on rest images compared to stress. The LV myocardial regions with ischemia, infarction, and those with RR patterns were defined according to the bull's eye display and the 17-segment model as recommended. Increased right ventricular uptake and dilated LV cavity were noted when present.

### Coronary Angiography and Echocardiographic Parameters

All patients underwent CA using the modified Seldinger technique for puncture. The use of the radial or femoral artery depended on the preference of the cardiologist performing the surgery. Coronary lesions were classified as nonobstructive, obstructive, significant, and chronic total occlusion when stenosis rates were < 50, 50 to 70, < 70, and 100% (chronic), respectively. Visual estimation was mainly used to determine the extent of coronary stenoses and functional assessment with fractional flow reserve was performed in selected lesions when in doubt.

A Philips IE33 system with a matrix array ultrasonographic transducer (X4.1 transducer; Philips Medical Systems, Bothell, Washington, United States) was used for echocardiographic measurements. Motion abnormalities were defined as hypokinetic, akinetic, or dyskinetic when present. LVEF was calculated by eyeball assessment with Simpson's method in selected cases.

### Statistical Analysis

Clinical and demographic characteristics are presented as mean ± standard deviation (SD) or median with interquartile range (IQR) for continuous variables, and frequency (percent) for categorical variables. The normality of the variables was determined using the Kolmogorov–Smirnov test with a Lilliefors significance correction and the Shapiro–Wilk test.

We used Spearman's rank correlation coefficient and Bland–Altman analysis for the agreement between LVEF derived from echocardiography and MPI data. We chose Spearman's rank correlation coefficient because it is a nonparametric measure of rank correlation, which assesses how well the relationship between two variables can be described using a monotonic function. This method was particularly appropriate for our data set, which did not follow a normal distribution. The Bland–Altman analysis was employed to assess the agreement between two quantitative measurements by constructing limits of agreement. The limits of agreement of the Bland–Altman plot were calculated with 95% confidence limits. This method was critical to understand the degree of agreement between LVEF values obtained from echocardiography and MPI and to identify any systematic bias. It provided a visual and quantitative approach to assess the consistency between these two diagnostic methods. Lastly, we used Fisher's exact test to investigate the association between the location of significant coronary stenoses on angiography and the presence of RR, ischemia, and infarction on MPI. This test was chosen because it is a precise test of the significance of the association between two categorical variables and is particularly useful when sample sizes are small. Given the categorical nature of our variables (location of coronary stenosis, RR, ischemia, and infarct), Fisher's exact test allowed us to accurately determine whether there were nonrandom associations between these variables.

A two-tailed *p* -value of < 0.05 was considered statistically significant. Data analysis was conducted using the IBM SPSS Statistics software (version 26; IBM Corporation, Armonk, New York, United States).

Approval was obtained from the local ethics committee and informed consent was waived because of the retrospective nature of the study (Approval number: 2023/06-16).

## Results

The primary study cohort was comprised of 67 patients (men 83%, aged 63.6 ± 10.5). The baseline characteristics of the study population are shown in Table 1 . About half of the study population had a history of some form of revascularization, and while 1 in 3 had experienced ACS, 46 patients (67.8%) had chronic coronary syndrome. The reported indications for MPI were as follows with decreasing frequency: Patients with stable angina who are unsuitable for exercise electrocardiogram (ECG) testing (i.e., unable to exercise due to physical disability, ventricular preexcitation, resting ST segment depression of more than 1 mm, left ventricular hypertrophy with ST-T abnormalities, digoxin use with associated ST-T abnormalities, and left bundle branch blocks on baseline ECG) and for investigation of ischemia after ACS.

**Table 1:** Baseline characteristics of the study population

	Total
Variable	( *n* = 67)
Demographics	
Age (y)	63.6 ± 10.5
Female	12 (17.9%)
Clinical history	
Acute coronary syndrome	22 (32.8%)
STEMI	15
NSTEMI	1
Unidentified ACS	6
Revascularization history	28 (41.7%)
Stent-only	16
CABG-only	10
CABG plus stent	2
Imaging parameters	
Echo LVEF (%) [median, (IQR)]	55 (20)
MPI stress LVEF (%) [median, (IQR)]	47 (18)
MPI rest LVEF (%) (mean ± SD)	51.1 ± 11.1
Comorbidities and risk factors	
Hypertension	44 (65.6%)
Diabetes	35 (52.9%)
Dyslipidemia	44 (65.6%)
Smoking	13 (19.4%)

Abbreviations: ACS, acute coronary syndrome; CABG, coronary artery bypass grafting; Echo, echocardiography; IQR, interquartile range; LVEF, left ventricular ejection fraction; MPI, myocardial perfusion imaging; STEMI, non-ST elevation myocardial infarction; SD, standard deviation; STEMI, ST elevation myocardial infarction.

**Fig. 2:**
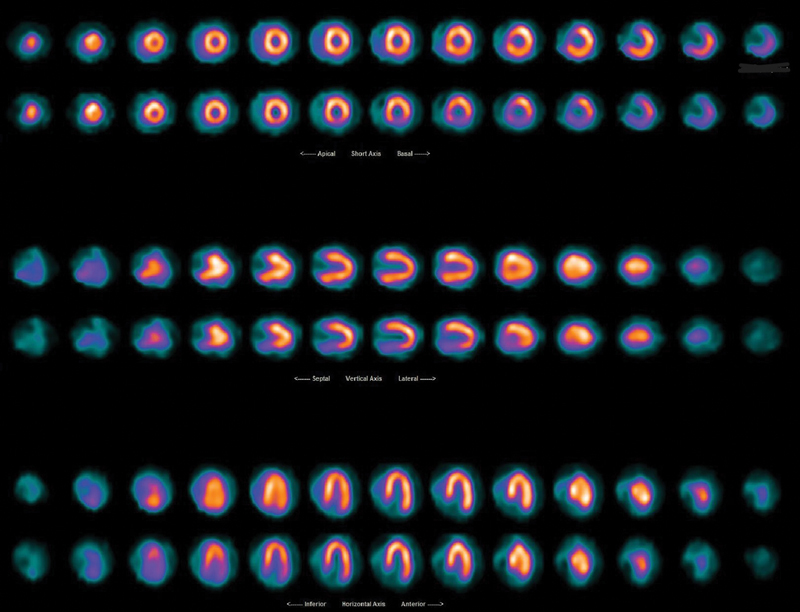
A myocardial perfusion scan from a patient with reverse redistribution (RR) pattern in inferior wall.

According to the results of the primary analysis, which included all 67 patients, including those with a history of CABG and those without CA, the RR pattern was most commonly seen in the inferior-posterior wall ( *n* = 41, 31.3%; see Fig. 2 ), followed by the apex ( *n* = 19, 14.5%) and anterior ( *n* = 12, 9.2%). In 40% of the patients ( *n* = 27), RR pattern was present in more than one region. Six patients (8.9%) had reversible defects and 16 (23.8%) patients had irreversible defects at separate regions than those seen with RR.

Although most of the physicians went on with invasive CA ( *n* = 40), 17 patients were treated only with medical anti-ischemic therapy, 2 patients were ordered coronary CT, and 2 underwent cardiac PET (with [18F]fluorodeoxyglucose) scan after a result of RR. Patients who received medical therapy alone had more history of CABG and previous revascularization compared to patients who underwent CA, coronary CT, and cardiac PET scan ( *p* = 0.02 and *p* = 0.02, respectively).

**Fig. 3:**
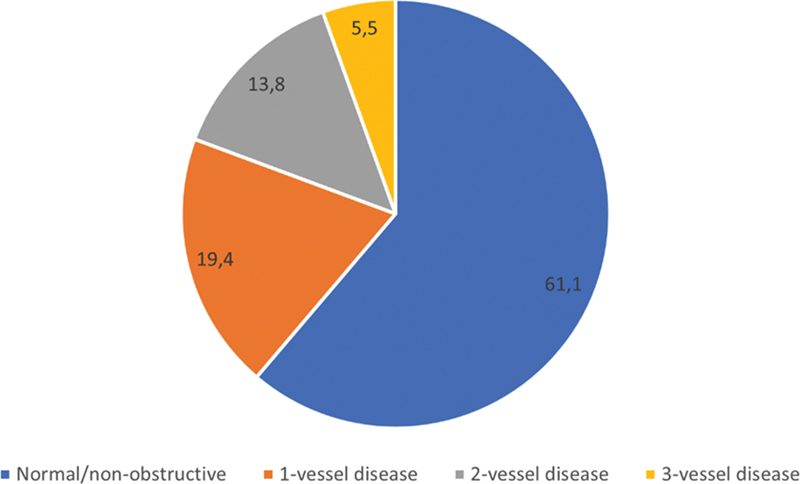
Coronary angiography results pie chart (percent).

**Fig. 4:**
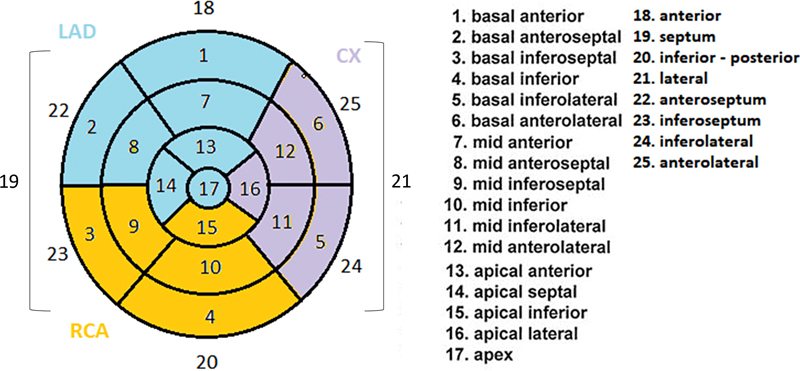
Twenty-five-segment model of myocardial segmentation (modified from the original 17-segment model).

A coronary angiogram was available for analysis in 36 of 67 patients. All patients undergoing invasive CA had RR with or without ischemia on MPI. Although most patients with RR pattern had normal/nonobstructive coronary angiograms (61.1%, *n* = 22), significant stenoses in 1, 2, and 3 vessels were present in 19.4% ( *n* = 7), 13.8% ( *n* = 5), and 5.5% ( *n* = 2) of patients, respectively ( Fig. 3 ). There was no difference in terms of gender, history of ACS or revascularization, and comorbidities between those with and without significant stenosis on CA. There was no correlation between the regions of the RR pattern and significant stenosis detected on CA ( *p* = 0.6, *p* = 0.5, *p* = 0.6, respectively, for left anterior descending artery, circumflex artery, and right coronary artery; see Fig. 4 for distribution of coronaries on a 25-segment model which was modified from the standard 17-segment model to better match scan results to the echocardiograms).

**Fig. 5:**
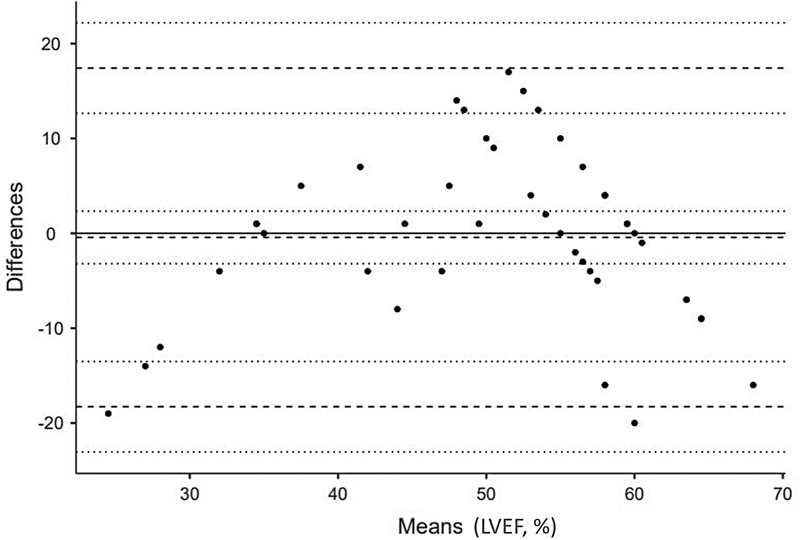
Bland–Altman plot illustrating the degree of correlation of left ventricular ejection fraction (LVEF) measured by myocardial perfusion imaging (MPI) versus echocardiography.

The linear regression analysis showed a correlation ( *r* = 0.74, *p* < 0.0001) between LVEF assessed with echocardiography and with MPI. The calculation of Bland–Altman limits of agreement with 95% confidence intervals showed that the lower limit was minus 18.2%, the upper limit was 17.4%, the mean difference was 1.1%, and 27 out of 29 measurements (93.2%) were within 1.96 SDs ( Fig. 5 ). A significant difference was found between stress and rest LVEF assessed by MPI (median [IQR]: 47 [18] vs. 51.5 [17], respectively; *p* = 0.007).

## Discussion

**Fig. 6:**
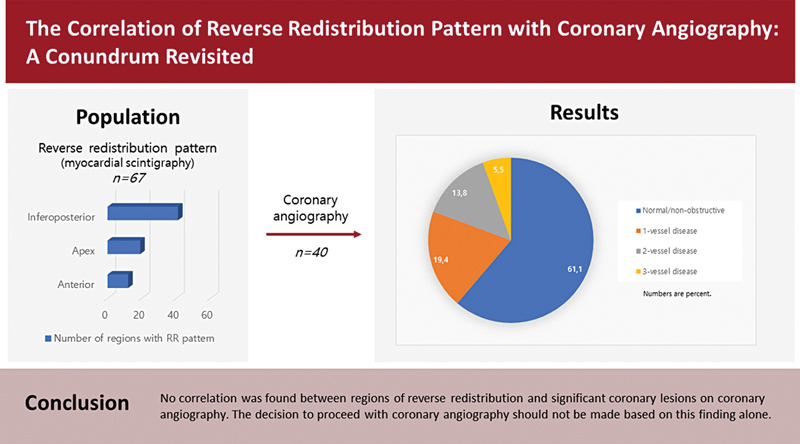
Central illustration.

This study, which was conducted to reevaluate the relationship between RR pattern and CA results, revealed that more than half of patients (61.1%) with RR pattern had normal/nonobstructive findings on CA (see Fig. 6 ).

The prevalence of the RR pattern in our study was 1.1%. Although there is a wide range of variation in the reported prevalence of RR pattern in the literature, most likely due to the diversity of settings in which the scans were performed, this result should be interpreted with caution because, given the nature of the 1-day study protocol, we may have missed a few of the RR patterns as only patients with abnormalities on the stress examination were called back for a rest examination.

In our study, the most common regions of the RR pattern were inferior-posterior, apex, and anterior, respectively. In three studies, the most common site of RR pattern was the anterior wall, while in one study RR was mostly seen in the inferior wall. 3 4 5 6 Consistent with the two previous studies, no RR pattern was found in the lateral wall in any patient. 5 6 It is possible that attenuation artifacts could account for some RR which is mostly found in the anterior (breast) and in the inferior (diaphragm) walls, but not in the lateral region.

There was a male predominance in our study population (82.1%, *n* = 55). This is in line with previous observations showing a similar male/female ratio. 7 8 In the context of comorbidities, the prevalences of diabetes, hypertension, and dyslipidemia were higher than observed in other studies (65, 52, and 65%, respectively). 3 7 8

Another important finding that supports other research is the high rates of ACS and revascularization history in patients with RR. 9 In contrast to earlier findings, however, no evidence of a relationship between areas of infarction and RR pattern was found. Further analysis revealed that the RR pattern was seen in 4 myocardial regions in 3 patients who had MI and revascularization in areas that do not have stenosis in repeat angiography. This finding seems consistent with other studies that found the same result and suggest that RR may be associated with epicardial artery patency and defects in microvascular perfusion. 10

The potential mechanisms behind this phenomenon are speculated to vary according to the settings in which they occur, such as acute or chronic coronary syndromes. For example, in the case of ACSs, reperfusion injury or persistent microvascular obstruction in the early phase postinfarction might lead to this pattern. 11 In the opposite situation with chronic coronary artery disease, the RR pattern was seen in patients with previous MI. 8 Therefore, in our opinion, it is possible that more than one factor may play a role in the formation of this pattern, either alone or in combination.

There was a modest agreement between resting LVEFs assessed by MPI and echocardiography. Consistent with the literature, this research found a statistically significant decrease in LVEF measured by MPI after pharmacological stress in all patients with RR. Poststress reduction in LVEF has been suggested as a high-risk finding associated with severe/extensive coronary disease in some studies, but not in others. 12 The difference lost significance in the secondary analysis of patients with known CA results in our study.

### Limitations

The findings in this report are subject to limitations as follows: First, because the study was a retrospective study, selection bias and confounding could not be eliminated. Furthermore, due to the lack of a control group, we could not make a comparison in many clinical situations. Second, the imaging system lacked attenuation correction, which could have resulted in false positives. Third, since no information on the body mass index of the patients was available in our study, it was not possible to investigate any possible artifactual errors arising from obesity. Finally, the relatively small and heterogeneous study population may have prevented more definitive conclusions.

## Conclusion

This study, which aimed to examine the relationship between RR patterns and coronary angiographic findings in the current era of widespread use of antiaggregant and anti-ischemic therapy, found no evidence of such a link. The second major finding was that 60% of the patients with RR pattern had normal/nonobstructive coronaries. Therefore, clinicians should not proceed to CA based on this finding alone. Instead, they should consider another noninvasive imaging modality, such as coronary CT, before proceeding to CA. Further research, especially in vitro, is needed to better understand the mechanisms underlying this particular phenomenon.
